# Deep neural networks detect suicide risk from textual facebook posts

**DOI:** 10.1038/s41598-020-73917-0

**Published:** 2020-10-07

**Authors:** Yaakov Ophir, Refael Tikochinski, Christa S. C. Asterhan, Itay Sisso, Roi Reichart

**Affiliations:** 1 The Hebrew University of Jerusalem, Jerusalem, Israel; 2 The Faculty of Industrial Engineering and Management, Technion—Israel Institute of Technology, Haifa, Israel

**Keywords:** Psychology, Computational science

## Abstract

Detection of suicide risk is a highly prioritized, yet complicated task. Five decades of research have produced predictions slightly better than chance (AUCs = 0.56–0.58). In this study, Artificial Neural Network (ANN) models were constructed to predict suicide risk from everyday language of social media users. The dataset included 83,292 postings authored by 1002 authenticated Facebook users, alongside valid psychosocial information about the users. Using Deep Contextualized Word Embeddings for text representation, two models were constructed: A Single Task Model (STM), to predict suicide risk from Facebook postings directly (Facebook texts → suicide) and a Multi-Task Model (MTM), which included hierarchical, multilayered sets of theory-driven risk factors (Facebook texts → personality traits → psychosocial risks → psychiatric disorders → suicide). Compared with the STM predictions (0.621 ≤ AUC ≤ 0.629), the MTM produced significantly improved prediction accuracy (0.697 ≤ AUC ≤ 0.746), with substantially larger effect sizes (0.729 ≤ *d* ≤ 0.936). Subsequent content analyses suggested that predictions did not rely on explicit suicide-related themes, but on a range of text features. The findings suggest that machine learning based analyses of everyday social media activity can improve suicide risk predictions and contribute to the development of practical detection tools.

## Introduction

Suicide is a leading cause of death worldwide and considerable scientific efforts are directed at early detection and prevention of suicide risk^1,2^. However, detecting suicide risk is a complex classification problem^3,4^. Findings from a recent meta-analysis of five decades of suicide research using traditional statistical methods showed that our ability to detect suicide risk from demographic, psychological, or medical factors is extremely limited. In fact, the prediction performances of suicide risk were “only slightly better than chance” (AUCs = 0.56–0.58)^5^. In the present work, we report on research showing that the combination of psychological knowledge, advanced machine learning techniques, and natural language processing (NLP) methods can considerably improve suicide risk predictions.

Machine learning techniques are becoming increasingly common in mental health research^6,7^. In a recent review of 300 mental health studies that employed machine learning/data driven algorithms, Shatte et al. concluded that such tools may improve our ability to detect and diagnose mental health conditions. However, the applicability of machine-learning models may still be limited because their predictions are often based on medical sources, such as neuroimaging data, counselling transcripts, and clinical reports. A recent study, for example, managed to develop a highly accurate suicide prediction model (0.769 ≤ AUC ≤ 0.792), based on the health records of patients who visited one of the Berkshire Health System hospitals^8^. Although valuable, these sources do not capture first-hand the patients’ natural behavior, nor do they include data from non-treated or non-diagnosed individuals.

Recent studies have therefore focused on ‘in the moment’, everyday exchanges collected from social media platforms^9,10^. The popularity of social media platforms, such as Facebook or Twitter, has created unprecedented opportunities to mine large data sets of everyday, user-generated content for patterns of communication that could be indicative of various mental health conditions. Research in this field has been particularly bountiful in the case of depressive disorders^11–13^.

In comparison, studies exploring suicide risk detection from user-generated social media posts are significantly fewer in number^14^. Together with earlier works on non-digital communication formats (e.g., written poetry or interviews)^15,16^, they show that natural language contains valuable signals that are indicative of suicide risk. However, we flag three general limitations of the existing research on suicide detection from social media activity:

First, offline, external validations of suicide risk are rarely included in the datasets. Instead, the ‘ground truth’ criterion for suicide risk is determined by *proxy diagnostic measures*, that is, judgements of suicidal thoughts or behaviors made by experts or non-experts based on the textual content posted by the users^14,17^. However, these judgments may not properly measure actual suicide risk (construct validity) due to self-presentation biases and language ambiguities on social media^18^. Moreover, text-based judgments will fail to identify users who do not choose to explicitly share suicidal or depressive feelings online^19^. Finally, in many cases, the data collection and judgment process has been conducted on postings from designated, suicide-related forums, such as “suicide watch” on Reddit^14,20^, thus limiting their applicability to more natural, everyday settings.

Second, existing research has mainly used text mining methods that are based on occurrence statistics of words that belong to pre-defined lexicons. However, the meaning of words (even the word ‘kill’) depend on their context. Moreover, natural language, especially in informal environments such as Facebook, may include non-words and emoji that are not listed in registered lexicons (e.g., Lolll, OMG,  ). Machine learning algorithms should therefore account for these unique language characteristics of social media.

Third, mental health conditions in general^21^ and suicide risk in particular^5^ are complex and heterogeneous phenomena with multiple genetic and environmental risk factors. Yet, the existing research on suicide and on other mental health manifestations in social media activities rarely considers the broader clinical picture of the condition. Predictions of suicide risk are expected to benefit from simultaneous computational analyses of multiple risk factors^3^.

### The present study

In this study, we leverage recent advancements in machine learning and construct deep neural network models to predict suicide risk from user-generated social media texts. A total of 1002 Facebook users completed a well-established, clinically valid screening tool of suicide risk^22^ and volunteered to disclose a year of their Facebook activity, resulting in a dataset of 83,292 postings.

Clinically validated data was collected on three sets of risk factors for suicide and for depressive episodes, which often precede suicidal behavior^23^. The first set comprised *psychiatric disorders* (internalizing psychopathology), the most severe risk factors for suicide behaviors^5,24^, including depression as well as generalized anxiety, which often appears in comorbidity with depression^23,25^. The second set included *psychosocial risks* for depression^26,27^, namely: depressive rumination, excessive worries^27,28^, feelings of loneliness, and lack of satisfaction with life^29,30^. The third and most distal set of factors included the Big Five *personality traits*^31^, since neuroticism and, to a lesser extent, extroversion have been associated with suicide behaviors^32^ and depression^23^.

Based on this dataset, we extracted representations of Facebook texts, using a deep Contextualized Word Embeddings (CWE) algorithm (see “Method” section). These text representations served as the input for two principal Artificial Neural Network (ANN) models, which were constructed for the purpose of the current study. The first model was a straightforward Single Task Model (STM) that aimed to predict suicide risk from users’ Facebook activity (Facebook texts → suicide). The second model was a Multi Task Model (MTM) that considered three additional, theory-driven layers of contributing factors (Facebook texts → personality traits → psychosocial risks → psychiatric disorders → suicide). We hypothesized that the MTM, which combines knowledge from both the psychological and the computational sciences, would significantly improve suicide risk prediction accuracy, compared to the STM and to the current state of suicide research. Finally, we provide interpretational analyses of the MTM predictions to identify textual features that may have contributed to the distinction between individuals with and without suicide risk.

## Method

### Tools and measurements

#### Suicide risk

Suicide risk was measured using the Columbia Suicide Severity Rating Scale (CSSRS)^22^. The CSSRS is considered a diagnostic tool of choice in clinical settings and empirical research, with high specificity and sensitivity^33,34^. The first part of the scale addresses passive suicide ideation^35^ (a wish to be dead and suicidal thoughts) and the second part addresses active suicide ideation and behaviors (suicidal thoughts with method, intent, or a specific plan and preparation to commit suicide). This second part was only presented to participants who reported suicidal ideation in the first part. This modular structure enables the extraction of two binary (yes/no) variables: a *general risk of suicide* (all the participants who had at least passive ideation) and a *high risk of suicide* (a sub-group of the 'general risk' participants who reported a specific method, intention, or plan to act on their suicidal thoughts). The scale does not address completed acts of suicide. In this study, the sum score of the CSSRS correlated with the sum scores of all other risk factors (see next) and especially with depression (*r* = 0.44), thus indicating a high convergent validity of the scale (Table 1).

**Table 1:** Descriptive statistics and correlations of the psycho-diagnostic measures (*N* = 1650).

	Suicide	Depression	Anxiety	Brooding	Worry	SWL	Lonely	Open	Conscientious	Extravert	Agreeable	Neurotic
Means (SD)	0.87 (1.41)	7.44 (5.94)	14.25 (5.7)	10.81 (3.53)	50.76 (15.59)	20.66 (8.14)	23.89 (6.73)	7.69 (2.03)	7.52 (1.88)	5.52 (2.40)	6.92 (2.01)	6.64 (2.40)
Depression	0.436**											
Anxiety	0.377**	0.754**										
Brooding	0.382**	0.610**	0.656**									
Worry	0.346**	0.552**	0.711**	0.656**								
SWL	− 0.360**	− 0.534**	− 0.449**	− 0.458**	− 0.423**							
Lonely	0.350**	0.572**	0.479**	0.539**	0.485**	− 0.607**						
Open	0.070*	− 0.019	0.033	0.047	0.033	− 0.012	− 0.044					
Conscientious	− 0.184**	− 0.303**	− 0.187**	− 0.254**	− 0.192**	0.269**	− 0.271**	0.100**				
Extravert	− 0.191**	− 0.242**	− 0.210**	− 0.187**	− 0.249**	0.273**	− 0.385**	0.143**	0.120**			
Agreeable	− 0.179**	− 0.262**	− 0.282**	− 0.207**	− 0.278**	0.262**	− 0.344**	0.053	0.113**	0.196**		
Neurotic	0.300**	0.486**	0.617**	0.564**	0.762**	− 0.393**	0.461**	− 0.025	− 0.272**	− 0.295**	− 0.281**	

Notice that the current research addressed low satisfaction with life whereas the SWL is formulated in a positive manner (i.e., high satisfaction with life). This positive formulation explains the negative correlation between SWL and depression.

*Suicide* the total score of the CSSRS, *SWL* satisfaction with life scale.

**p < 0.01.

#### Risk factors for suicide and depression

Major depressive disorder was measured using the Patient Health Questionnaire-9 (PHQ-9)^36^. Generalized anxiety disorder was measured using the GAD-7 scale^37^. Depressive rumination (brooding) was measured using five items from the Ruminative Responses Scale (RSS)^38^. Excessive worrying was measured using the Penn State Worry Questionnaire (PSWQ)^39^. Loneliness was measured using the 10-item version of the UCLA-Loneliness Scale^40^. Low satisfaction with life was measured using the Satisfaction With Life Scale (SWLS)^41^. Personality traits were assessed using the short version of the Big Five Inventory (BFI-10)^42^. Descriptive statistics and zero-order correlations of all psycho-diagnostic measures are provided in Table 1. Detailed descriptions of measures are provided in the Supplementary Material.

### Sample and dataset

The procedures of the study comply with the ethical standards of the Helsinki Declaration of 1975, as revised in 2008. All procedures were approved by the Ethics for Research on Human Subjects Committees of the Technion, Israel Institute of Technology and the Hebrew University of Jerusalem. Informed consent was obtained from all participants. Participant recruitment was conducted through Amazon’s Mechanical Turk (MTurk). A strict data quality assurance protocol for online data collection was applied^43^. This included a method to screen out bogus participants and eight attention checks (see Supplementary Material).

After reading and signing the consent form, participants completed eight psycho-diagnostic measures, and gave us a one-off authorization to download their Facebook posts up to 12 months prior to the research date. The Facebook data was extracted to an encrypted data storage through a designated application, which was developed for the purpose of the current study. Upon completion, participants who met the criterion for suicide risk received a letter that included a list of mental health services and an encouragement to seek help (see Supplementary Material).

The initial sample included 2,685 English speaking adult US residents. From this sample, we excluded 236 users with suspicious IP addresses, 464 users who did not pass the attention checks, and 335 users who did not publish any Facebook postings. We then extracted a total of 85,643 textual postings that were generated and posted by the remaining 1650 users. However, since valuable ANN-based predictions require sufficient amount of text, we further excluded users who did not reach the *median* number of Facebook posts, which was calculated to be 10 postings per profile. The final dataset included 83,292 posts generated by 1002 Facebook users (23.25% male) who published at least 10 posts. The average number of postings per user was 82.35 (*SD* = 106.79) and the average number of words in each post was 31.14 (*SD* = 66.56). Table 2 presents the socio-demographic characteristics (age, gender, and income) of the current sample.

**Table 2:** Socio-demographic characteristics of users at general risk and of non-suicidal users (*N* = 1002).

	No risk (*SD*)	General risk (*SD*)	Statistics *p* value	Effect size Cohen's *d* [95% CI]
Number of participants	641 (63.97%)	361 (36.03%)		
Mean number of posts	74.1 (97.9)	97.0 (119.9)	*t*(1000) = 3.27**	0.207 [0.083,0.332]
Mean age	38.3 (11.0)	34.6 (10.6)	*t*(1000) = − 5.09***	− 0.322 [− 0.448, − 0.197]
Gender (%male)	23.4%	23.0%	*χ*^2^ = 0.005, *p* = 0.945	
Annual income in US dollars	58,563 (36,627)	48,389 (35,526)	*t*(998) = − 4.26***	− 0.270 [− 0.396, − 0.145]

*No risk* users who did not report of any suicidal ideation, *General risk* all the users who reported of at least passive ideation in the suicide scale.

**p < 0.01, ***p < 0.001.

The collected Facebook posts were then matched with the psycho-diagnostic information and the suicide risk scores of the 1002 users who generated these posts. Based on previous studies that investigated the unique characteristics of MTurk samples, we note that the prevalence of mental health issues, and especially of major depression, is significantly higher in MTurk, compared with the general population^43–45^. Correspondingly, relatively high rates of suicide risk were found in the current sample: 361 users (36.03%) met the criterion for ‘*general risk of suicide*,’ of which 132 (13.17%) met the criterion for *high risk of suicide*. Users at general risk differed from users who did not report of any suicidal ideation on several socio-demographic parameters (Table 2). They were younger (*M* = 34.6, *SD* = 10.6, *t* =  − 5.09, *p* < 0.001) and relatively poorer (*M* = 48,389$, *SD* = 35,526 *t* =  − 4.26, *p* < 0.001) compared with non-suicidal users (mean age = 38.3, *SD* = 11, mean annual income = 58,563$, *SD* = 36,627). At risk users also had more Facebook postings (*M* = 97, *SD* = 119.9) than non-suicidal users (*M* = 74.1, *SD* = 97.9), *t* = 3.27, *p* < 0.01. The magnitude of all socio-demographic differences was small (0.21 ≤ Cohen's *d* ≤ 0.32). Gender differences between suicidal and non-suicidal users were not significant (χ^2^ = 0.005, *p* = 0.945).

### ANN-based models

**Figure 1:**
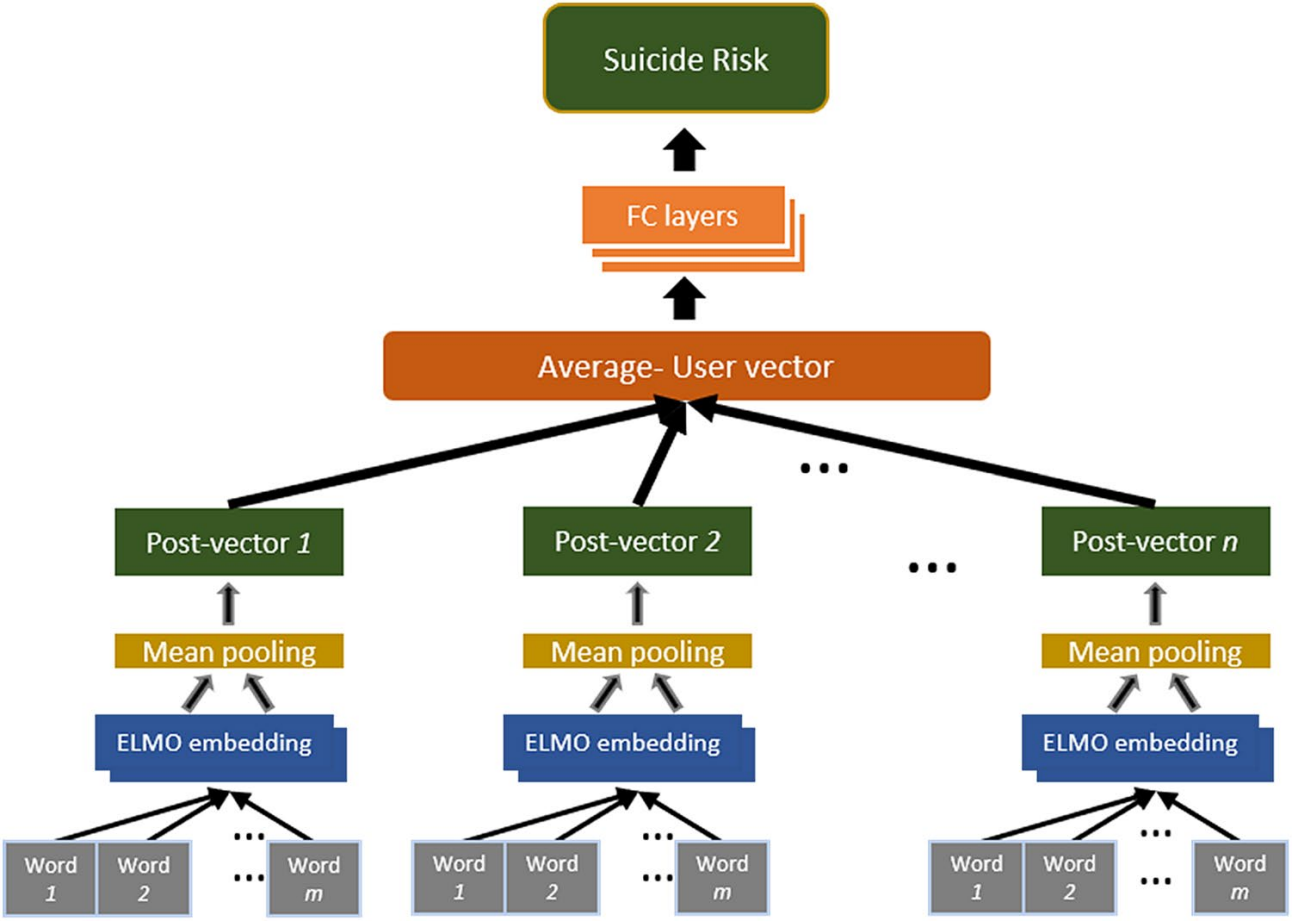
The single task model (STM). *FC layers* fully connected layers.

**Figure 2:**
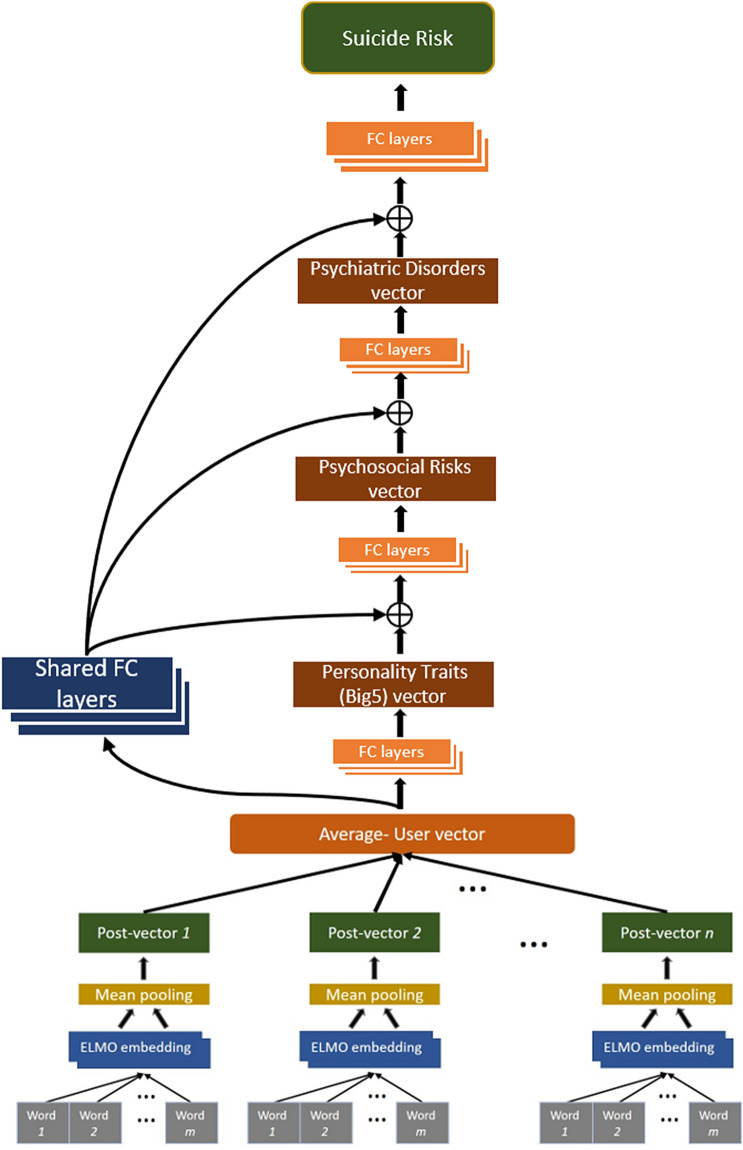
The multi task model (MTM). *FC layers* fully connected layers; The sign ⊕ symbolizes the vector concatenation operator.

In order to predict suicide risk from Facebook texts, two ANN-based models were constructed (see Figs. 1, 2). The construction process included four steps: *First*, we extracted representations of the Facebook texts, using ELMo, a deep Contextualized Word Embeddings (CWE) algorithm. *Second*, we created the two types of ANN models (STM and MTM), in which the ELMo based representations served as inputs. *Third*, we trained and optimized the models to predict suicide risk from (represented) Facebook texts. *Finally*, we tested the quality of the predictions made by both models. Following is a detailed description of the process.

Both models consisted of identical input and output layers. The input consisted of representations of Facebook texts, which are 1024-dimensional vectors extracted by ELMo, a state-of-the-art method for Embeddings from Language Models^46^. ELMo comprises a deep language model through multiple bi-directional Long-short-Term-Memory (LSTM) layers. The use of ELMo has two advantages over other text representation techniques, such as word count or N-grams: It is character-based (rather than word-based) and therefore it generates representations also to non-words (i.e. words that do not appear in formal dictionaries), which are popular in social media language (e.g., Lolll or OMG) and it generates representations of words within their context (i.e., a given word receives different representations depending on its surrounding text).

Using a pre-trained ELMo model (available at https://tfhub.dev/google/elmo/2), we extracted a 1024-dimensional embedding vector for each Facebook post in our data through mean-pooling over the contextualized word embeddings generated for the post. The overall textual-activity of the user was represented as the average of its post vectors and served as input to the ANN models.

The output of the two models consisted of a single binary (yes/no) variable of suicide risk. Following the modular structure of the suicide scale, we considered two variants of each model, one for predicting *general risk of suicide* and one for predicting *high risk of suicide*. The two variants of the Single Task Model (STM) were constructed to predict suicide risk directly from textual contents of Facebook posts, using a set of fully-connected layers (textual content → suicide). The two Multi Task Model (MTM) variants were constructed to predict a hierarchical combination of multiple factors. We integrated three sets of auxiliary risk factors that could mediate the link between Facebook postings and suicide risk (textual content → personality traits → psychosocial risks → psychiatric disorders → suicide). An illustration of these risk factors is provided in the Supplementary Material, Fig. A.

Each auxiliary layer is accompanied by a set of fully-connected layers, thus forming several “subnetworks.” The subnetwork located at the bottom of the model (i.e., the Personality traits) is activated directly by the input layer (Facebook content), while the subnetworks at the middle (Psychiatric disorders and Psychosocial risks) are activated by the previous subnetwork’s output, which is concatenated with outputs from a shared set of fully-connected layers. The shared set of layers is activated directly from the input layer (i.e., the textual representations), which allows the subnetworks to get direct information from the input layer (and not just from the previous subnetwork). This architecture introduces inductive bias to the suicide prediction model through the auxiliary tasks, while learning a shared set of parameters for the multiple tasks in order to reduce the risk for overfitting. Finally, the Suicide layer at the top of the model is activated by the output generated by the Psychiatric disorders layer and by the output of the shared set of hidden layers (Fig. 2). The loss functions of both models are provide in the Supplementary Material.

The dataset was randomly divided into three portions, such that the percentage of users at general suicide risk is identical in all portions. In the learning phase, each ANN-based model was trained on the Facebook texts of 70% of the users in the sample (701 users), to classify users as being suicidal or not. Each learning example was comprised of the Facebook posts of one participant together with the suicide label of that participant (positive/negative for general/high suicide risk). For the MTM model, each learning example also included the auxiliary variable scores of the participant (i.e., their scores on the three sets of psychodiagnostics scales).

In the model development phase, a hyper-parameter tuning process was conducted on another 15% of the data (150 users). In this phase, we also considered several alternative models that were more complicated than the STM but less complicated than the MTM. These partial models included one of the MTM three auxiliary layers (e.g., psychiatric disorders) but their detection performance did not reach the quality of the complete MTM. Finally, in the test phase, the algorithm was used to provide suicide risk predictions to the remaining 15% of the dataset (151 users) based on their Facebook postings only (that is, the MTM did not have access to the personality, psychosocial, or psychiatric external labels).

This learning process was conducted five times, each time with a different random division of the dataset to three parts, to prevent from overfitting and selection bias. In each division, we maintained the same composite of users and postings. The results from this fivefold cross-validation process are presented in the following section. The full details of the models, including their objective function, training algorithm, hyper-parameters, and tuning procedure are provided in the Supplementary Material.

## Results

### Detection performance of suicide risk

A receiver operating characteristic curve (ROC curve), which plots the True Positive prediction rates of each model against its False Positive prediction rates was generated and the Area Under the ROC Curve (AUC) was calculated. AUC provides a reliable estimation of the quality of the predictions across all possible classification thresholds. It specifically suits class imbalanced tasks in which the positive class (suicidal users) is significantly smaller than the negative class (non-suicidal users)^47^. AUC scores can also be easily transformed to Cohen’s *d*, the most common effect-size measure in experimental psychology^48^.

Table 3 presents the prediction performance of the two models. The average prediction performances of the STM was significantly higher than chance level (AUC of 0.5), both for general risk [AUC = 0.621, 95% CI: 0.576, 0.657] and for high suicide risk [AUC = 0.629, 95% CI 0.606, 0.660]. A transformation of the AUC scores to effect sizes^48^ indicated a medium effect size for general risk (Cohen’s *d* = 0.436) and high risk (Cohen’s *d* = 0.466) of suicide. The results from the STM therefore suggest that single task models may produce higher than chance predictions that are comparable, and perhaps even superior, to previously documented non-machine learning suicide detection efforts^5^.

**Table 3:** Detection performance (average AUC scores) of the STM and MTM (*N* = 1002).

Task	General suicide risk		High suicide risk	
Model	STM	MTM	STM	MTM
Average AUC scores	0.621	0.746	0.629	0.697
95% confidence interval	576, 0.657	0.727, 0.765	0.606, 0.660	0.690, 0.707

*STM* single task model, *MTM* multiple tasks model, *AUC* area under the receiver operating characteristic curve, *Average AUC scores* the average scores of the five AUC scores that were obtained in the cross-validation analyses.

Importantly, the inclusion of all risk factors in the MTM produced substantial improvements in prediction accuracy for general [AUC = 0.746, 95% CI 0.727, 0.765] and high suicide risk [AUC = 0.697, 95% CI 0.690, 0.707]. These predictions showed a medium-to-large effect size for high suicide risk (Cohen’s *d* = 0.729) and a large-to-very large effect size for general suicide risk (Cohen’s* d* = 0.936). The absence of an overlap between the Confidence Intervals of the STM and the MTM, indicates that the observed improvements in the prediction quality of the MTM is significant. On average, the MTM produced higher AUC scores than the STM, both in the general risk case (mean difference = 0.124, 95% CI 0.074, 0.162) and the high risk case (mean difference = 0.064, 95% CI 0.034, 0.090).

A similar pattern of results was found when the Facebook texts were represented with the recent attention-based BERT model (Bidirectional Encoder Representations from Transformers)^49^. The comparison between the prediction performances with BERT and ELMo (see Supplementary Material, Table A) indicated that the observed patterns and predictions extend beyond the specific CWE method (ELMo) that was employed in this study. The results support our hypothesis that a multilayered prediction model consisting of all three layers of contributing factors (Facebook content → personality traits → psychosocial risks → psychiatric disorders → suicide) would demonstrate improved predictions, in comparison with a single task model and with the previous efforts in the literature to predict suicide risk without machine learning and natural language processing (NLP) methods.

### Interpretation of the observed predictions

In order to gain a deeper insight into what could be the specific textual indications that allowed the machine to make predictions, we applied the following procedure to the fold in which the MTM achieved its best general risk AUC score. We first transformed the continuous general risk score that each participant received from the MTM to a binary general suicide risk label (positive/negative). The threshold from the ROC curve that was chosen for this transformation was the one that returned the maximum ratio between the True Positive and the False Positive rates. Based on this threshold, users were classified into four groups: True Positive, False Positive, True Negative, and False Negative (a plain language description of these classes is available in the Supplementary Material).

We then conducted a word search for explicit suicide-related content among users at general risk who were classified correctly by the MTM (*N* = 33 True Positive users, 22% of the test data). Specifically, we searched for morphological variations of three words: Suicide, Kill, and Die. This search produced eight mentions of *suicide*, 20 mentions of *kill*, and 44 appearances of *die*. Notably, only in a single instance did these words appear in messages directly related to suicide. Two typical examples are “my back is killing me” and “It’s gonna be a good Halloween, probably going to die, but it’ll be fun.” Even in the case of the most explicit phrase “I want to die,” the full context was: “Cramps so bad, I want to die”.

Finally, we applied *Term Frequency Inverse Document Frequency (TF-IDF)* analysis^50^ to detect specific words that distinguish between True Positive and True Negative users. For each one of the above four classes, we extracted the 100 most characteristic words that best distinguished the given class from the rest (see Supplementary Material, Table B. The results indicated that the most distinctive words of the True Positive class (i.e., users at general suicide risk who were identified correctly) consisted of negatively charged words (bad, worst), including swear words (bitch, fucking), words referring to feelings of distress (mad, cry, hurt, sad), and to physical complaints (sick, pain, surgery, hospital). Similar to the previous analysis, explicit suicide-related words, such as kill, die, or suicide did not appear in this list.

In contrast, the most distinctive words of the True Negative class (i.e., non-suicidal users who were identified correctly) consisted of positive words (great, happy, perfect), including positive emotions (loving, love, peace) and events (wedding, thanksgiving), positive experiences of belonging and friendships (together, friends, mother, wife), and positive attitude towards life (blessed, gift, wishes). Interestingly, a dominant theme in the postings of these True Negative users was religion and spirituality (Christ, church, God, faith). Taken together, these findings suggest that the ANN model did not rely on explicit suicide manifestations, but on a wide range of textual features, including emotionally charged topics.

## Discussion

Suicide is a leading cause of death worldwide^1^ and early detection and prevention of suicide risk is a cross-national mission. However, five decades of suicide research have yielded prediction performances that are only marginally better than chance^5^. In the present study, we leveraged recent advancements in NLP methods to predict suicide risk from textual features of everyday, user-generated social media posts.

The results from the STM indicate that textual features of Facebook postings predict both general and high suicide risk (0.621 ≤ AUC ≤ 0.629). The observed medium effect size of the STM-based results is comparable, and perhaps even superior to earlier suicide prediction attempts that did not use machine learning^5^. This is a noteworthy proof of concept, given that the predictors of suicide risk in this study were not extracted from medical sources or demographic information, but from everyday user-generated behavior in a naturalistic environment (social media).

More importantly, the results confirmed our expectation that the MTM, which integrated multiple theory-driven risk factors, would produce improved prediction accuracy of suicide risk from textual social media postings (0.697 ≤ AUC ≤ 0.746), compared with the STM and with the existing literature on suicide risk prediction^5^. In this research, the MTM produced medium-to-large and large effect sizes for high and general suicide risk, respectively. These high-quality predictions were significantly better than the STM-based predictions. Altogether, these results demonstrate the potential of machine learning and NLP methods for the detection of externally validated suicide risk from everyday social media behavior, as well as the importance of integrating theory-driven factors when using such methods.

### Theoretical contributions to research on suicide risk prediction from social media

The present work builds on earlier attempts to predict suicide risk from social media by incorporating several improvements. First, the incorporation of theory-driven measures strengthened the construct and external validity of the findings. In contrast to previous works that used proxy diagnostic measures (i.e., judgements made by experts or non-experts based on users' textual content) as the ground truth for suicide risk^20,51^, the current study relied on external, clinically valid measure of suicide risk. The inclusion of additional psycho-diagnostic tools (i.e., the personality, psychosocial, and psychiatric measures) contributed to the validity of the study as well.

Second, the dataset on which the prediction algorithms were developed was extracted from everyday (inter)actions in a non medical environment, rather than from a medical source or even from a designated online suicide-support forum, thus extending the generalizability of the findings to multiple and ordinary settings. To maintain this high ecological validity without compromising the internal validity of the psychological information, a strict data quality assurance protocol was applied, and only valid responses were included. In addition, post hoc internal reliability and convergence validity checks were conducted on all variables (see Supplementary Material). These procedures, along with the externally obtained psychodiagnostics measures, contributed to the construction of a large and high quality dataset, compared to existing research in this field^9^.

Third, to our knowledge, this study is the first to apply state-of-the-art artificial neural networks and deep CWEs for text representation in order to predict suicide risk from social media. The use of ELMo has two advantages over other text representation techniques, such as word count or N-grams: It generates representations also for non-words, which are popular in social media language (e.g., Lolll or OMG), and for words within their context (i.e., a given word receives different representations depending on its surrounding text). Complementing the advantages of CWE methods, the application of ANN-based models provided an effective platform for learning multiple variables jointly^52^, thus enabling the analysis of a multilayered psychosocial profile of suicidal and non-suicidal individuals.

Fourth, the combination of the four main features of the study (i.e., a CWE representation method, ANN modeling, psychodiagnostic measures, and analysis of everyday language) enabled the extraction of valuable language usage patterns that could not be hypothesized a priori. Since many users refrain from sharing explicit depressive content^19,53^, algorithms that rely solely on explicit distress-related content or established lexicons are arguably more susceptible to False Negative results. In contrast, the current ANN models were capable of detecting subtler cues of mental health difficulties. In fact, our word search for explicit suicide references revealed that the majority of the True Positive (suicidal) users rarely posted content that directly referred to suicide ideation. Correspondingly, the TF-IDF analysis did not reveal explicit suicide-related words either.

Although interpretations remain speculative, the TF-IDF outcomes suggest that correct classifications of suicide risk could be based on the appearance of negatively charged words (swearing, distress, physical complaints). These negative themes are in line with previous work on the digital footprints of depression in social media^12^. Interestingly, in this study, the high quality performance of the models may also result from the distinct language of the True Negative (non-suicidal) users, which included references to positive emotions and experiences, positive attitudes towards life, as well as religion and spirituality. This interpretation is in line with previous work emphasizing the protective role of meaning in life and religious/community involvement against actual suicide behaviors^54^. This finding provides another reason to employ ANN models, given that lexicon-based models that aim to discover explicit suicide-related content might miss these subtler signals (e.g., religious expressions in the True Negative group). ANN models may detect suicide risk even when users refrain from sharing explicit, suicide-related content.

### Limitations of the current research

The first limitation of the present work concerns the self-report nature of the psycho-diagnostic data collection procedure. Although usage of such screening tools is common in large-scale mental health surveys, it may be less accurate than face-to-face, structured clinical interviews or formal medical assessments of suicide risk (or related psychiatric disorders). This study also did not investigate actual suicide deaths (completed suicide), which can be seen as the ultimate predictive criterion for suicide. Yet, in this study, we chose well-established psycho-diagnostic measures and ensured the quality of the self-reported responses by using multiple validation checks (internal reliability, convergence validity, and a data quality assurance protocol; see Supplementary Material). Nevertheless, we recommend that future research includes additional forms of external criteria for suicide risk assessment.

A second limitation concerns the focus on language-based input to the ANN models. A recent study on depression detection indicated the superiority of textual contents over other types of social network signals, such as length or timestamps of postings^12^. It is possible however, that the incorporation of non-textual social media activity features (e.g., pictures) could further improve the quality of suicide risk predictions.

A third limitation concerns the socio-demographic makeup of our sample. Compared with a recent US national survey conducted by the Centers for Disease Control and Prevention (CDC)^55^, the participants in the current sample were more likely to be younger, female, and have a slightly higher average annual income (see Table 2). In addition, although we specifically sought to recruit individuals from the general population, the current sample consisted of relatively high rates of suicide risk and emotional distress. These rates seem to characterize users of crowdsourcing platforms (such as MTurk), even when rigorous data quality measures are implemented^43,44,56^, and may therefore limit the generalizability of the findings. Finally, as our predictive models were text-based, Facebook users who had published less than 10 posts in 12 months had to be excluded from the analyses. The current findings (and text-based models in general) may therefore be less relevant for users who rarely publish textual content on social media.

### Implications of the current research

The integration of machine learning methods in mental health practices seems to be a promising avenue for advancing personalized psychiatry practices^6^ and improving detection and diagnosis efforts of complex psychiatric phenomena^7,57^. The knowledge gained from this study and from similar studies^9^ could lay the foundations for the development of practical monitoring tools capable of tracking and analyzing cues from online communication, automatically and unobtrusively. Ideally, such applications would integrate cues from several information streams (including medical records) and alert individuals, family members, or mental health caregivers, when increased levels of suicide risk are detected.

A second implication of the current study relates to computational mental health research. We join previous recommendations to investigate the prediction performances of machine learning methods ‘in the wild’^6^, including everyday environments, such as ubiquitous social media platforms. Based on the current findings, we recommend that such endeavors combine state-of-the-art computational techniques along with theory-driven components from clinical and social sciences. While this study did not include every known risk factor, it anchored the predictions of suicide risk within the theoretical framework of the multifaceted nature of suicide^2^. We evidenced significant improvements in suicide risk predictions from social media postings when the detection algorithms incorporated the wider clinical picture of suicide and its related risk factors. In the present study, this progress was made possible due to a close collaboration between computational, social, and clinical scientists. Genuine, multi-disciplinary collaboration seems to be a prerequisite for the field of computational mental health research to make significant progress.

## Supplementary information

Supplementary Information.

## Data Availability

The data of this study are available on request from the corresponding author [YO]. The data are not publicly available due to their containing information that could compromise the privacy of research participants.
