# Supplementary material for: Deep neural networks detect suicide risk from textual facebook posts
Source: Sci Rep. 2020 Oct 7;10:16685. doi: 10.1038/s41598-020-73917-0 (PMC7542168; doi:10.1038/s41598-020-73917-0)
Supplement: Supplementary file 1 — Supplementary Information. [file 41598_2020_73917_MOESM1_ESM.docx]

**Supplementary Material**

**Deep Neural Networks Detect Suicide Risk from Textual Facebook Posts**

Yaakov Ophir,^1,2^ Refael Tikochinski,^1,2^ Christa Asterhan,^1^ Itay Sisso,^1^ Roi Reichart^2^

^1^The Hebrew University of Jerusalem; ^2^Technion – Israel Institute of Technology

**Psycho-diagnostic Tools**

**Suicide risk.** Suicide risk was measured using the well-established Columbia Suicide Severity Rating Scale (CSSRS)^1^. The CSSRS was originally developed to help clinicians structure their clinical interviews and assess the existence and severity of suicide risk with high levels of accuracy. The scale demonstrated high sensitivity and specificity scores in suicide prediction and it is considered a “diagnostic tool of choice,” both in clinical settings and in empirical research^2,3^. Upon consultation with the principal developer of the CSSRS (Posner, personal written communication), we chose to administer the electronic self-report version of the scale, in light of the fact that the current research examined participants from a crowdsourcing platform. The electronic version of the CSSRS has been demonstrated to have psychometric validity and prediction accuracies that are comparable to the original clinician version of the scale^4,5^.

The scale consists of six binary (yes/no) items that are presented to the participants in two parts. In the first part, participants were asked to complete Item 1 that addressed a “wish to be dead” *(“Have you wished you were dead or wished you could go to sleep and not wake up?”*) and Item 2 that addressed “suicidal thoughts” (*“Have you actually had any thoughts of killing yourself?”*). Only if participants answered “yes” to item #2 on suicidal thoughts, they were then exposed to the second part of the scale that examined the severity of the risk. Item 3 addressed suicidal thoughts with method (*“Have you been thinking about how you might kill yourself?”*). Item 4 addressed suicidal intent (*“Have you had these thoughts and had some intention of acting on them?”*). Item 5 addressed suicide intent with specific plan (*“Have you started to work out or worked out the details of how to kill yourself? Do you intend to carry out this plan?”*), and Item 6 addressed actual suicide behaviors (*“Have you ever done anything, started to do anything, or prepared to do anything to end your life?”*). Participants who answered “yes” to this last item were then asked to indicate when they engaged in such behavior (over a year ago, between three months and a year ago, or within the last three months).

The modular structure of the CSSRS enables the extraction of two binary (yes/no) variables: a *general risk of suicide* (participants who met the criterion of the first part of the scale, that is answering “yes” to item 2) and a *high risk of suicide* (a sub-group of participants at suicide risk who also responded “yes” to at least one of the items in the second part of the scale). The total sum score of the “yes” answers to all six items serves as another indication for the severity of the suicide risk. In this study, the total score of the CSSRS was positively correlated with all the examined risk factors (see Table 1 of the main text) and especially with depression (*r* = 0.44), thus indicating a high convergent validity of the scale.

**Major Depressive Disorder (MDD).** Major depression was measured using the Patient Health Questionnaire-9 (PHQ-9)^6^, a nine-item scale that targets the nine symptoms of depression described in the DSM. Each item (symptom) is scored from 0 to 3 (not at all, several days, more than half the days, and nearly every day). Given its well-established validity and high sensitivity and specificity^7^, the PHQ-9 is preferred over all other screening tools for depression^8^. The internal consistency of the scale in the current sample was high (α = .90) and the correlation with suicide total scores was high (*r* = 0.44).

**Generalized Anxiety Disorder (GAD).** GAD was measured using a well-established, seven-item scale named GAD-7^9^. Each item, scored from 0 to 3 (not at all, several days, more than half the days, and nearly every day), targets one of the seven symptoms of the disorder. The total score of the scale (range 0 – 21) serves as an indication for both the existence and the severity of the disorder. The internal consistency of the scale in the current sample was high (α = .92). The evidenced comorbidity between GAD and major depression as indicated in a bivariate Pearson, was very high (*r* = 0.75).

**Depressive rumination (brooding).** Depressive rumination as mentioned above is a maladaptive pattern of thinking in which people focus on their depressive feelings and enter a repetitive loop of negative thoughts^10,11^. Specifically, the unconstructive component of this ruminative thinking, which has been shown to be strongly associated with depression was named “brooding”^12^. Brooding was measured using five items rated from 1 (almost never) to 4 (almost always) from the frequently used Ruminative Responses Scale (RRS)^13^. Respondents read a general statement about depressive events (“*People think and do many different things when they feel depressed*”) and are asked to indicate to what extent they engage in a given response. An example for a brooding response is: “*Think about a recent situation, wishing it had gone better*.” The internal consistency of the 5 brooding items, in the current sample was good (α = .82) and the correlation with depression was high (*r* = 0.61).

**Excessive worrying.** A second pattern of negative thinking is excessive and subjectively uncontrollable worries about the future^14^. To assess excessive worrying patterns, we used the Penn State Worry Questionnaire (PSWQ)^15^. The PSWQ is a well-established research tool^16^ that comprises 16 items, rated on a five-point scale (1 = not as all typical of me, 5 = very typical of me). The items address various aspects of pathological worry including its excessiveness (e.g., “*Many situations make me worry*”) and the subjective feeling of uncontrollability (e.g., “*Once I start worrying, I cannot stop*”). The internal consistency of the PSWQ in the current sample was high (α = .96) and the correlation with depression was high (*r* = 0.55).

**Loneliness.** Experiences of loneliness were measured using the 10-item version of the UCLA-Loneliness Scale^17^. The items, rated from 1 (Never) to 4 (Always), encompass various aspects of loneliness experiences (e.g., “*How often do you feel that you lack companionship*”). This version of the scale demonstrated high levels of convergent validity and internal consistency (Elphinstone, 2018). The internal consistency of the scale in the current sample was high (α = .92) and the correlation with depression was high (*r* = 0.57).

**Low satisfaction with life.** The general sense of satisfaction with life was measured using the Satisfaction With Life Scale (SWLS)^18^. This short scale comprises five items, rated from 1 (strongly disagree) to 7 (strongly agree). All items are formulated in a positive manner (e.g., “*The conditions of my life are excellent*”). Although we were interested in low satisfaction with life, we kept the original positive style of the scale to “break” the overall negative atmosphere of the research and to promote participants’ attentiveness along the research. The SWLS demonstrated good psychometric characteristics^19^ and moderate-strong negative relationships with depression^20,21^. The internal consistency of the scale in the current sample was high (α = .93) and the negative correlation of this positive scale with depression was high (*r* = -0.53).

**Personality traits.** Personality traits were assessed using the short version of the Big Five Inventory (BFI)^22^. The BFI-10 includes ten items that target the five clusters of personality traits, originally formulated in the standard 44-item BFI: Extraversion, Neuroticism, Openness to Experience, Agreeableness and Conscientiousness^23^. Each trait in the BFI-10 is measured by only two items that are rated from 1 (disagree strongly) to 5 (agree strongly). The BFI-10 achieved high levels of reliability and validity^22^ and is currently widely used in research settings. Consistent with the literature on depression, the correlation between the personality trait of neuroticism and depression was high (*r* = 0.51).

The convergent validity of the psychosocial scales was high. As expected, the total score of the suicide scale was positively correlated with all the risk factors examined in the study and especially with depression (*r* = 0.49). Consistent with the literature on depression described above, the comorbidity between depression and anxiety was very high (*r* = 0.75) and the four psychosocial risk factors (i.e., brooding, excessive worry, loneliness, and low satisfaction with life) were strongly correlated with depression (Pearson’s *r* ranging from 0.53 to 0.61). The personality trait of neuroticism was also strongly correlated with depression (*r* = 0.49).

**Ethical Considerations**

Crowdsourcing-based suicide research involves an ethical challenge: how to safeguard the well-being of suicidal participants, without the possibility of face-to-face interactions? To address this ethical challenge, the first author (a clinical psychologist) cooperated with a consortium of experts to develop an online suicide research protocol. Prior to consenting to participate in the study, participants were informed that if their responses would indicate some form of suicidal risk, we would contact them through the data collection platform. Each participant who met the CSSRS criterion for general suicide risk (i.e., suicidal thoughts with or without a specific method or a concrete plan) then received a designated letter in which we encouraged them to seek help and provided them with a list of available “hotlines” and national mental health services. The complete description of the protocol and the ethical considerations made in the current research are available by the first author upon request.

**Data Quality**

In light of recent concerns regarding the quality of crowdsourcing-based data, we applied a newly developed rigid data quality assurance protocol^24^. The inclusion criteria were: having a Facebook account and having previous proven experience in MTurk-based studies. Proven experience was defined as past completions of at least 100 MTurk tasks, with a minimum of 95% success rate. To avoid bogus responses, we limited the participation to US residents and excluded users with suspicious Internet Protocol (IP) addresses. To ensure the quality of the unsupervised responses, we implemented a designated inattentiveness scale that comprised eight hidden attention checks. These checks included four types of data-quality measurements (i.e., “infrequency items,” “time measurements,” “person-total correlation,” and “long string analysis”), which were embedded in the various self-report scales of the study^24^.

**Loss functions of the ANN-based Models**

As illustrated in Figure 1 of the main article, the Single-Task Model (STM) consisted of an input and an output layer, which are connected by a set of fully-connected layers. In contrast, the Multi-Task Model (MTM) contained three additional hierarchically organized auxiliary layers: Facebook content → personality traits → psychosocial risks → psychiatric disorders → suicide risk.

The loss function of the STM models is the *binary cross-entropy*:

$${1. \mathcal{L}}_{suicide}= -\frac{1}{N}\sum_{i=1}^{N} y_{i}\log\left( p\left( y_{i} \right) \right)+\left( 1- y_{i} \right)\log\left( 1- p\left( y_{i} \right) \right)$$

Where *N* is the number of training examples, $y_{i}$ indicates whether participant *i* belongs to the suicide group ($y_{i}=1$) or not ($y_{i}=0$) according to the ground truth, and ${p(y}_{i})$ indicates the probability of $y_{i}$ as predicted by the model.

The loss function of the MTM is the sum of the output layer’s and the auxiliary layers’ loss functions:

$$2. \mathcal{L=}\mathcal{L}_{suicide}+\mathcal{L}_{aux}$$

Where $\mathcal{L}_{suicide}$ is the binary cross-entropy loss function like before, and $\mathcal{L}_{aux}$ is the sum of all *mean squared errors* (MSEs) calculated for each of the auxiliary variables in the set *A*={Depression, Anxiety, Brooding, Worry, SWL, Lonely, Open, Conscientious, Extravert, Agreeable, Neurotic}:

$${3. \mathcal{L}}_{aux}= \sum_{a\in A} -\frac{1}{2N}\sum_{i=1}^{N} \left( y_{i}^{\left( a \right)}-\hat{y}_{i}^{\left( a \right)} \right)^{2}$$

where *N* is the number of training examples, $y_{i}^{\left( a \right)}$ is a continuous variable representing the ground truth score of the auxiliary-variable *a* for subject *i*, and $\hat{y}_{i}^{\left( a \right)}$ is the predicted score for this variable according to the model.

**ANN-based Models – Parameter Estimation (Learning)**

The optimization of the model was conducted with batch sub-gradient descent (batch-size of 32), using the back-propagation algorithm^27^ and the RMSProp optimizer^28^ with a momentum parameter of 0.9. The hyper-parameters of the models were tuned using a grid-search method on the development data, and the model with the highest development data AUC score for suicide risk (general or high) was selected. These hyper-parameters included the number of fully connected layers {1, 2, 3} (note that all the sub-networks of the MTM had the same number of fully connected layers), the number of neurons in each layer {16, 32, 64, 128, 256, 512, 1024}, and the type of the activation function {*hyperbolic tangent*, *sigmoid*}. The hyper-parameters of the optimization algorithm were the learning rate {0.001, 0.005, 0.01, 0.05}, and the number of epochs {1000, 2500, 5000}.

Following, we describe the selected hyper-parameters of the STM and the MTM of the first fold of the 5-folds, cross validation process. All other hyper-parameters are available upon request from the authors. The selected hyper-parameters of the General risk STM included: 3 fully connected layers, 32 neurons, an activation function of *hyperbolic tangent*, a learning rate of 0.01 and 2,500 epochs. The selected hyper-parameters of the high risk STM included the same parameters but with 5,000 epochs.

The selected hyper-parameters of the general risk MTM included: 2 fully connected layers, 16 neurons, an activation function of *Sigmoid hyperbolic tangent*, a learning rate of 0.001 and 5,000 epochs. The selected hyper-parameters of the high risk MTM: 3 fully connected layers, 16 neurons, an activation Sigmoid function, a learning rate of 0.001 and 1,000 epochs.

**Four Possible Classes of Suicide Risk Predictions**

The continuous general risk score that each participant received from the MTM (the best performing model) was transformed into a binary general suicide risk label (positive/negative). The threshold from the ROC curve that was chosen for this transformation was the one that returned the maximum ratio between the True Positive and the False Positive rates. Based on this threshold, users were classified into four groups: True Positive, in which a suicidal user is correctly detected (true) by the model as suicidal (positive); False Positive, in which a non-suicidal user is incorrectly detected (false) as suicidal (positive); True Negative in which a non-suicidal user is correctly determined (true) as not suicidal (negative); and False Negative in which a suicidal user is incorrectly determined by the model (false) as non-suicidal (negative).

**Figure A. Illustration of the hierarchical "pyramid" of risk factors for suicide**

Note: The bottom of the proposed pyramid consists of the big five personality traits (i.e., openness; conscientious; extraversion; agreeableness; and neuroticism). The middle layers consist of the psychosocial risk factors (i.e., depressive rumination, worries, loneliness, and low satisfaction with life) and the psychiatric disorders (i.e., depression and anxiety), and the top layer consists of the predicted output, which is the two types of binary suicide variables (i.e., general and high suicide risk).

**Suicide detection using BERT**

Table A provides a comparison of the results of the STM and MTM models, between the case where the text representation is made by ELMo (the text representation method that was employed in the main study) and the case where the text is represented by the recent attention-based BERT model (Bidirectional Encoder Representations from Transformers)^29^. The similarities between the two cases included an equivalent range of AUC scores and improved predictions of the theory-driven MTM. On average, the MTM produced higher AUC scores than the STM, both in the general risk case (Mean difference = .073, 95% CI: .030, .139) and the high risk case (Mean difference = .076, 95% CI: .053, .100). The difference between the two cases included better BERT performance on the high risk group compared to the general risk group (an opposite phenomenon is observed with ELMo). The overall similar patterns indicate that the main conclusions of the research, and particularly the one about the importance of theory-driven multi-task modeling for suicide risk prediction, are independent of the specific text representation method employed by the model.

**Table A.** Detection performance of the models using BERT Task

|  | General suicide risk | | High suicide risk | |
| --- | --- | --- | --- | --- |
| Model | STM | MTM | STM | MTM |
| Average AUC scores using ELMo | .621  95% CI: .576, .657 | .746  95% CI: .727, .765 | .629  95% CI: .606, .660 | .697  95% CI: .690, .707 |
| Average AUC scores using BERT | .599  95% CI: .569, .660 | .672  95% CI: .608, .708 | .679  95% CI: .638, .727 | .744  95% CI: .676, .780 |

Note: STM = Single Task Model; MTM = Multiple Tasks Model; AUC = Area Under the receiver operating characteristic Curve; Average AUC scores = The average scores of the five AUC scores that were obtained in the cross-validation analyses.

**Table B.** Term Frequency Inverse Document Frequency (TF-IDF)

The following table presents the 100 most characteristic words that best distinguished each one of the four classes from the others in the general risk MTM of the fold (from the 5-folds cross validation process) that resulted in the best AUC score.

|  | **True Positive** | **False Positive** | **True Negative** | **False Negative** |
| --- | --- | --- | --- | --- |
| 1 | anymore | president | lord | fight |
| 2 | lose | husband | shall | ufb |
| 3 | kinda | season | blessed | office |
| 4 | probably | games | king | daddy |
| 5 | mad | gun | gift | fire |
| 6 | poor | strong | james | system |
| 7 | positive | wonder | christ | email |
| 8 | room | cat | church | experience |
| 9 | pretty | continue | missed | nobody |
| 10 | sitting | update | drink | shows |
| 11 | top | news | loving | ufd |
| 12 | cheese | damn | anybody | just |
| 13 | air | paid | god | america |
| 14 | eating | writing | monday | war |
| 15 | sick | none | wedding | none |
| 16 | okay | words | faith | questions |
| 17 | pain | talking | christmas | king |
| 18 | cry | change | jesus | ya |
| 19 | actually | ass | coffee | etc |
| 20 | instead | hot | prayer | pray |
| 21 | seriously | cold | however | like |
| 22 | easy | test | prayers | day |
| 23 | bit | watched | version | bus |
| 24 | reason | within | history | prayers |
| 25 | game | children | kill | one |
| 26 | clothes | fb | wishes | can |
| 27 | mother | asking | answer | happy |
| 28 | daughter | knew | state | know |
| 29 | hurt | grow | comment | get |
| 30 | worst | cancer | law | whats |
| 31 | bad | blood | important | will |
| 32 | account | shit | heaven | time |
| 33 | felt | red | mental | five |
| 34 | theyre | yesterday | father | texas |
| 35 | wow | dad | dr | sister |
| 36 | enjoy | holiday | ready | soul |
| 37 | lady | country | son | running |
| 38 | cut | near | pass | street |
| 39 | sleep | look | spirit | coming |
| 40 | fuck | loved | ufc | green |
| 41 | supposed | weeks | email | yeah |
| 42 | bed | looked | peace | american |
| 43 | pizza | funny | line | fine |
| 44 | quite | text | thanksgiving | along |
| 45 | gets | around | perfect | attention |
| 46 | guess | support | ufb | close |
| 47 | drive | relationship | war | human |
| 48 | door | course | save | died |
| 49 | thinking | putting | lol | company |
| 50 | surgery | wanted | together | problems |
| 51 | gonna | seems | dear | aint |
| 52 | literally | found | thank | business |
| 53 | thats | couple | just | prayer |
| 54 | sleeping | world | great | share |
| 55 | bitch | daily | sunday | area |
| 56 | cream | john | happy | listen |
| 57 | heart | looks | working | bit |
| 58 | wonderful | ask | fall | water |
| 59 | big | several | day | truly |
| 60 | arent | posts | cause | missing |
| 61 | might | moving | st | go |
| 62 | fucking | half | given | character |
| 63 | hospital | age | today | page |
| 64 | told | seeing | help | people |
| 65 | sad | company | men | store |
| 66 | doesnt | longer | love | love |
| 67 | couldnt | kept | choose | local |
| 68 | wall | months | friends | group |
| 69 | favorite | tv | brother | learned |
| 70 | taking | florida | others | retweeted |
| 71 | cleaning | high | holy | send |
| 72 | stupid | others | time | busy |
| 73 | nap | away | like | good |
| 74 | ugh | given | city | gonna |
| 75 | start | kids | everyone | song |
| 76 | entire | women | giving | now |
| 77 | brain | place | roll | anybody |
| 78 | car | hand | please | im |
| 79 | wear | sit | florida | park |
| 80 | times | weekend | mother | sorry |
| 81 | dinner | hey | copy | see |
| 82 | play | fear | question | pass |
| 83 | story | run | wife | gas |
| 84 | Isnt | voice | know | birthday |
| 85 | calling | dark | friend | wishes |
| 86 | white | called | forget | asking |
| 87 | spent | spend | cleaning | end |
| 88 | mind | eyes | teacher | books |
| 89 | online | learn | get | lives |
| 90 | hopefully | light | group | miss |
| 91 | ice | history | pray | everyone |
| 92 | making | true | child | jesus |
| 93 | rest | saw | can | missed |
| 94 | feet | body | go | dark |
| 95 | order | heard | known | figure |
| 96 | cute | open | safe | wedding |
| 97 | understand | happened | one | entire |
| 98 | type | move | busy | today |
| 99 | sure | write | american | came |
| 100 | summer | due | lets | forget |

Note: This table presents the 100 most characteristic words that best distinguished each one of the four classes (True Positive, True Negative, False Positive, and False Negative), from the others, using *Term Frequency Inverse Document Frequency (TF-IDF)*.

**References**

1 Posner, K. *et al.* The Columbia–Suicide Severity Rating Scale: initial validity and internal consistency findings from three multisite studies with adolescents and adults. *American Journal of Psychiatry* **168**, 1266-1277 (2011).

2 Drapeau, C. W. *et al.* Screening for suicide risk in adult sleep patients. *Sleep Medicine Reviews* **46**, 17-26, doi:<https://doi.org/10.1016/j.smrv.2019.03.009> (2019).

3 Weber, A. N., Michail, M., Thompson, A. & Fiedorowicz, J. G. Psychiatric emergencies: assessing and managing suicidal ideation. *Medical Clinics* **101**, 553-571 (2017).

4 Mundt, J. C. *et al.* Feasibility and validation of a computer-automated Columbia-Suicide Severity Rating Scale using interactive voice response technology. *Journal of psychiatric research* **44**, 1224-1228 (2010).

5 Viguera, A. C. *et al.* Comparison of electronic screening for suicidal risk with the Patient Health Questionnaire Item 9 and the Columbia Suicide Severity Rating Scale in an outpatient psychiatric clinic. *Psychosomatics* **56**, 460-469 (2015).

6 Kroenke, K., Spitzer, R. L. & Williams, J. B. W. The PHQ-9: Validity of a Brief Depression Severity Measure. *Journal of General Internal Medicine* **16**, 606-613, doi:10.1046/j.1525-1497.2001.016009606.x (2001).

7 Spitzer, R. L., Kroenke, K. & Williams, J. B. W. Validation and utility of a self-report version of PRIME-MD: the PHQ primary care study. *Jama* **282**, 1737-1744 (1999).

8 El-Den, S., Chen, T. F., Gan, Y.-L., Wong, E. & O’Reilly, C. L. The psychometric properties of depression screening tools in primary healthcare settings: A systematic review. *Journal of Affective Disorders* **225**, 503-522, doi:<https://doi.org/10.1016/j.jad.2017.08.060> (2018).

9 Spitzer, R. L., Kroenke, K., Williams, J. B. W. & Löwe, B. A brief measure for assessing generalized anxiety disorder: The GAD-7. *Archives of Internal Medicine* **166**, 1092-1097, doi:10.1001/archinte.166.10.1092 (2006).

10 Nolen-Hoeksema, S., Wisco, B. E. & Lyubomirsky, S. Rethinking rumination. *Perspectives on Psychological Science* **3**, 400-424, doi:10.1111/j.1745-6924.2008.00088.x (2008).

11 Mor, N. & Winquist, J. Self-focused attention and negative affect: A meta-analysis. *Psychological Bulletin* **128**, 638-662, doi:10.1037/0033-2909.128.4.638 (2002).

12 Schoofs, H., Hermans, D. & Raes, F. Brooding and reflection as subtypes of rumination: Evidence from confirmatory factor analysis in nonclinical samples using the Dutch Ruminative Response Scale. *Journal of Psychopathology and Behavioral Assessment* **32**, 609-617 (2010).

13 Nolen-Hoeksema, S. & Morrow, J. A prospective study of depression and posttraumatic stress symptoms after a natural disaster: The 1989 Loma Prieta earthquake. *Journal of Personality and Social Psychology* **61**, 115-121, doi:10.1037/0022-3514.61.1.115 (1991).

14 Brown, T. A., Antony, M. M. & Barlow, D. H. Psychometric properties of the Penn State Worry Questionnaire in a clinical anxiety disorders sample. *Behaviour research and therapy* **30**, 33-37 (1992).

15 Meyer, T. J., Miller, M. L., Metzger, R. L. & Borkovec, T. D. Development and validation of the penn state worry questionnaire. *Behaviour research and therapy* **28**, 487-495 (1990).

16 Fresco, D. M., Mennin, D. S., Heimberg, R. G. & Turk, C. L. Using the Penn State Worry Questionnaire to identify individuals with generalized anxiety disorder: A receiver operating characteristic analysis. *Journal of behavior therapy and experimental psychiatry* **34**, 283-291 (2003).

17 Russell, D. W. UCLA Loneliness Scale (Version 3): Reliability, validity, and factor structure. *Journal of personality assessment* **66**, 20-40 (1996).

18 Diener, E., Emmons, R. A., Larsen, R. J. & Griffin, S. The Satisfaction With Life Scale. *Journal of Personality Assessment* **49**, 71-75, doi:10.1207/s15327752jpa4901_13 (1985).

19 Pavot, W. & Diener, E. in *Assessing well-being* 101-117 (Springer, 2009).

20 Blais, M. R., Vallerand, R. J., Pelletier, L. G. & Brière, N. M. L'échelle de satisfaction de vie: Validation canadienne-française du" Satisfaction with Life Scale.". *Canadian Journal of Behavioural Science/Revue canadienne des sciences du comportement* **21**, 210 (1989).

21 Schimmack, U., Oishi, S., Furr, R. M. & Funder, D. C. Personality and life satisfaction: A facet-level analysis. *Personality and social psychology bulletin* **30**, 1062-1075 (2004).

22 Rammstedt, B. & John, O. P. Measuring personality in one minute or less: A 10-item short version of the Big Five Inventory in English and German. *Journal of research in Personality* **41**, 203-212 (2007).

23 John, O. P. & Srivastava, S. in *Handbook of personality: Theory and research (2nd ed.)* (ed L. A. Pervin O. P. John) 102-138 (Guilford Press, 1999).

24 Ophir, Y., Sisso, I., Asterhan, C. S. C., Tikochinski, R. & Reichart, R. The turker blues: Hidden factors behind increased depression rates among Amazon’s Mechanical Turkers. *Clinical Psychological Science* **8**, 65-83 (2020).

25 Peters, M. E. *et al.* Deep contextualized word representations. *arXiv preprint arXiv:1802.05365* (2018).

26 Pennington, J., Socher, R. & Manning, C. D. 1532-1543.

27 Goodfellow, I., Bengio, Y. & Courville, A. *Deep learning*. (MIT press, 2016).

28 Tieleman, T. & Hinton, G. Lecture 6.5-rmsprop: Divide the gradient by a running average of its recent magnitude. *COURSERA: Neural networks for machine learning* **4**, 26-31 (2012).

29 Devlin, J., Chang, M.-W., Lee, K. & Toutanova, K. Bert: Pre-training of deep bidirectional transformers for language understanding. *arXiv preprint arXiv:1810.04805* (2018).
